# Molar Distalization by Clear Aligners with Sequential Distalization Protocol: A Systematic Review and Meta-Analysis

**DOI:** 10.3390/jfb15060137

**Published:** 2024-05-21

**Authors:** Christie Shen, Tiffany H. Park, Chun-Hsi Chung, Chenshuang Li

**Affiliations:** 1 Department of Orthodontics, School of Dental Medicine, University of Pennsylvania, Philadelphia, PA 19104, USA; chrishen@upenn.edu (C.S.); chunc@upenn.edu (C.-H.C.); 2 School of Dental Medicine, University of Pennsylvania, Philadelphia, PA 19104, USA; thaparkk@upenn.edu

**Keywords:** molar distalization, clear aligners, sequential distalization, anchorage loss, orthodontics

## Abstract

Introduction: With the popularity of clear aligners, the sequential distalization protocol has been more commonly used for molar distalization. However, the amount of molar distalization that can be achieved, as well as the accompanying side effects on the sagittal dimension, are unclear. Methods: Registered with PROSPERO (CRD42023447211), relevant original studies were screened from seven databases (MEDLINE [PubMed], EBSCOhost, Web of Science, Elsevier [SCOPUS], Cochrane, LILACS [Latin American and Caribbean Health Sciences Literature], and Google Scholar) supplemented by a manual search of the references of the full-reading manuscripts by two investigators independently. A risk of bias assessment was conducted, relevant data were extracted, and meta-analysis was performed using RStudio. Results: After the screening, 13 articles (11 involving maxillary distalization, two involving mandibular distalization) met the inclusion criteria. All studies had a high or medium risk of bias. The meta-analysis revealed that the maxillary first molar (U6) mesiobuccal cusp was distalized 2.07 mm [1.38 mm, 2.77 mm] based on the post-distalization dental model superimposition, and the U6 crown was distalized 2.00 mm [0.77 mm, 3.24 mm] based on the post-treatment lateral cephalometric evaluation. However, the U6 mesiobuccal root showed less distalization of 1.13 mm [−1.34 mm, 3.60 mm], indicating crown distal tipping, which was validated by meta-analysis (U6-PP angle: 2.19° [1.06°, 3.33°]). In addition, intra-arch anchorage loss was observed at the post-distalization time point (U1 protrusion: 0.39 mm [0.27 mm, 0.51 mm]), which was corrected at the post-treatment time point (incisal edge-PTV distance: −1.50 mm [−2.61 mm, −0.39 mm]). Conclusion: About 2 mm maxillary molar distalization can be achieved with the sequential distalization protocol, accompanied by slight molar crown distal tipping. Additional studies on this topic are needed due to the high risk of bias in currently available studies.

## 1. Introduction

Achieving effective and efficient molar distalization to correct molar relationships and create space for crowding relief has been a long-lasting subject of debate in orthodontics. Historically, some of the most commonly used maxillary molar distalization strategies include inter-arch appliances such as class II elastics [1], Carriere 3D Motion appliance [2], Forsus fatigue resistant device [3], mandibular anterior repositioning appliance (MARA) [4], Herbst appliance [5], SAIF-springs [6], as well as intra-arch appliances such as the Pendulum [7], Jones jig [8], First Class Appliance [9], and Distal Jet [10]. Other distalization strategies include extraoral appliances such as headgear [11]. For many of the appliances mentioned above, there are concerns about patients’ acceptance [12]. For instance, several patients have reported difficulty in eating soon after delivery of these fixed appliances [13]. Other side effects reported by patients include toothaches, limited maximum opening, aching jaws, difficulty in the upkeep of oral hygiene, and soreness on the lip/cheek due to abrasion from appliances [12,14].

In the past two decades, clear aligners have become popular as a more esthetic, less invasive, and more hygienic option for patients seeking orthodontic treatment [15]. To achieve molar distalization with clear aligners, the sequential distalization protocol was introduced [16]. The sequential distalization protocol is designed to start with moving the second molar distally. When the second molar reaches 50% of the total movement, the first molar starts moving distally, and so on up to the canine, and lastly, en masse incisor retraction is initiated. During the whole protocol, inter-arch elastics are often used to utilize the opposing arch as the anchorage. However, whether this protocol is a viable option for a significant amount of molar distalization is still questionable [17,18]. When evaluated on the crown level on dental models, some articles reported up to 2.68 ± 0.50 mm distalization of the mesial buccal cusp of the maxillary first molar [19,20,21,22,23], while other articles reported unsatisfactory amounts of distalization were achieved [24,25]. Patterson et al. found that anterior–posterior correction achieved at the end of clear aligner therapy for class II patients is only 6.8% of the predicted amount (0.23 mm achieved of the 3.29 mm predicted amount) [24], and both Patterson et al. [24] and Taffarel et al. [25] concluded that treatment of Class II malocclusions with clear aligners would not meet the standards of the American Board of Orthodontics (ABO) Model Grading System.

It is worth noting that while bodily distalization is a more desirable treatment outcome with molar distalization, several molar distalization strategies report crown distal tipping and mesial out–distal in rotation of molars [19,20], which are unwanted side effects that easily cause relapse [26]. The previous literature indicates that pure crown tipping is the most predictable movement of clear aligners [17], and the movement involving any amount of root control has poor predictability, especially for the posterior teeth [17,27]. Thus, whether the sequential distalization protocol in clear aligner therapy can achieve bodily distalization of the molars needs to be further evaluated.

In addition, with inter-arch elastic usage as an integral part of the sequential distalization protocol, anchorage loss in opposing arches has been reported in conjunction with molar distalization [28]. Excessive incisor proclination or protrusion can result in undesirable side effects such as alveolar bone loss and gingival recession [29]. These concerns suggest the need to evaluate the amount of anchorage loss created by molar distalization.

Considering all the clinical questions mentioned above, a systematic review was conducted in the current study to examine the current evidence on the amount of molar distalization achievable with the sequential distalization protocol of clear aligner therapy. Moreover, this review aims to assess the accompanying effects of rotation and tipping of molars as well as anterior anchorage loss, ultimately providing clinical insight into the effectiveness and limitations of clear aligner therapy.

## 2. Materials and Methods

This study was registered with PROSPERO (registration number: CRD42023447211) on 1 August 2023, and is compliant with the 2020 Preferred Reporting Items for Systematic Reviews and Meta-Analyses (PRISMA) guideline [30]. All original articles were accessed through a search from the following electronic databases: MEDLINE (PubMed), EBSCOhost, Web of Science, Elsevier (SCOPUS), Cochrane, LILACS (Latin American and Caribbean Health Sciences Literature), and Google Scholar, with an initial search finish date of 13 February 2024.

### 2.1. Study Selection Criteria

**Figure 1:**
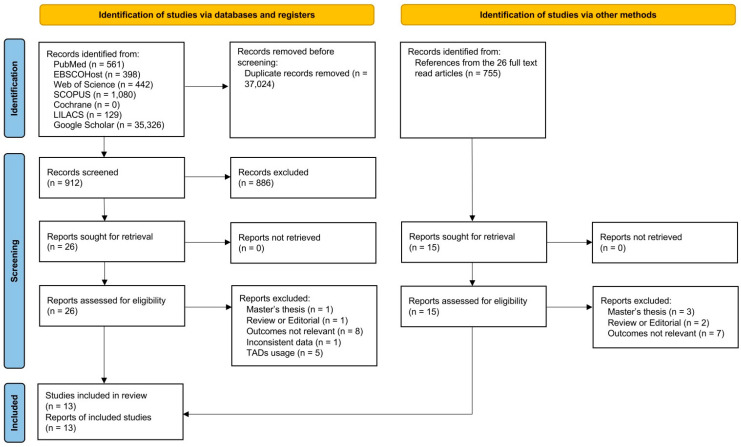
The PRISMA flow diagram demonstrating the study identification and screening.

Based on the framework of population, interventions, comparison, and outcomes (PICOs), we conducted a systematic literature review on the sequential distalization protocol of clear aligner therapy for molar distalization (Table 1). The inclusion criteria comprised (1) longitudinal studies (both prospective and retrospective) comparing pre- and post-distalization/treatment records, (2) participants with permanent dentition, and (3) molar distalization achieved by sequential distalization protocol. The exclusion criteria were (1) participants with congenital abnormalities or systemic pathologies, (2) case reports, (3) conference abstracts, (4) opinions, editorials, guidelines, or letters to the editors, (5) systematic reviews, (6) utilization of TADs or other auxiliaries during molar distalization, (7) molar distalization protocol not described, and (8) inconsistent data within the article and did not receive responses from the corresponding author(s). No language or date restrictions were imposed. The PRISMA flow diagram illustrating the process of obtaining the final included articles is presented in Figure 1.

### 2.2. Search Strategy

Our search strategy in all included databases is as follows: (“aligners” AND “molar distalization”), (“aligner” AND “molar distalization”), (“clear aligners” AND “molar distalization”), (“clear aligner” AND “molar distalization”), (“sequential distalization”), (“class II” AND “aligners”), (“class II” AND “aligner”), (“class II” AND “clear aligner”), (“class II” AND “clear aligners”), (“class III” AND “aligners”), (“class III” AND “aligner”), (“class III” AND “clear aligner”), (“class III” AND “clear aligners”), and (“invisible removable thermoplastic appliance”). In addition to the initial search, we manually reviewed the references cited in the articles identified for full-text reading. The full texts of these articles were thoroughly examined and evaluated against the predetermined inclusion and exclusion criteria. Two authors (C.S. and T.H.P.) independently carried out the literature search and screening to ensure the reliability and comprehensiveness of the results. In cases of discrepancies between the two authors, a third author (C.L.) was consulted for further discussion.

### 2.3. Data Extraction and Analysis

For all the articles included for further data analysis, relevant information was extracted from each article, including study type, arch treated, sample size, gender, age, clear aligner brand, type of records, timing of treatment records, as well as parameters evaluating molar distalization, molar rotation, molar tipping, and maxillary and mandibular anterior anchorage loss reported by dental model superimposition or by radiographic analysis.

### 2.4. Risk of Bias/Quality Assessment

Due to the heterozygosity of the study types of the included studies, the risk of bias protocol was modeled after one established in a previous publication [31], which is similar in design to our study. A total of 17 biases were evaluated in four categories: study design, study measurements, statistical analysis, and others (Table 2), which were scored by two authors (C.S. and T.H.P.) individually. In cases of disagreement, a third author (C.L.) provided input. Each article’s score was calculated by dividing the number of met criteria by the total number of criteria. The risk of bias—low, medium, or high—was determined based on randomization and reliability testing. A low risk of bias was assigned if both reliability and randomization criteria were met. A high risk of bias was indicated if inter-rater reliability was not assessed and if randomization was not conducted. All other studies were categorized as having a medium risk of bias (Table 2).

### 2.5. Statistical Analysis

The outcomes of this study were as follows: (1) the amount of molar distalization achieved during distalization with clear aligners, as well as the amount of concurrent (2) molar rotation, (3) molar tipping, and (4) anterior anchorage loss. A meta-analysis utilizing the data extracted from the included articles was conducted using RStudio (version 2023.09.1+494, Posit Software, PBC) [40,41]. In cases where articles only provided the mean difference along with upper and lower 95% confidence intervals, the standard deviation was computed using the conventional definition $SD=\sqrt{N}\times (upper\;limit-lower\;limit)/3.92$, regardless of the normal distribution within the sample population [42]. The meta-analysis was carried out employing a random effects model, and heterogeneity was evaluated for variance among studies using the Tau2 method (τ²). The results were presented as mean and 95% confidence interval [CI]. Sensitivity analysis and selective reporting within studies were not evaluated due to the limited number of studies included per analyzed variable.

## 3. Results

### 3.1. Literature Searching and Study Selections

Through the initial search with seven electronic databases, 37,936 potential articles (561 from PubMed, 398 from EBSCOHost, 442 from Web of Science, 1080 from SCOPUS, 0 from Cochrane, 129 from LILACS, and 35,326 from Google Scholar) were identified (Figure 1). A total of 912 articles remained for abstract screening following duplicate removal. A total of 26 articles were retrieved for full-text reading. A total of 755 records were also manually retrieved from the references of the full-text reading articles and 15 additional articles were retrieved for full-text reading. A total of 28 of the retrieved articles were excluded because they were master's theses [43,44,45,46], reviews or editorials [47,48,49], inconsistent data [50], utilized TADs [51,52,53,54,55], or the outcomes were not relevant [18,25,27,56,57,58,59,60,61,62,63,64,65,66,67]. Therefore, after adhering to the guidelines presented by the PRISMA, 13 articles were included for final analysis [19,20,21,22,23,32,33,34,35,36,37,38,39].

### 3.2. Risk of Bias

The strength of evidence was evaluated by conducting a methodological risk of bias assessment on the 13 studies included (Table 2). Among these, only one study [32] reported both random sampling and random allocation of treatment. The remaining studies did not provide information on randomization. Four studies [32,33,35,39] mentioned blinding conducted by the examiner, but only one [32] of them extended blinding to the statistician. Other studies did not incorporate blinding measures. Intra-rater reliability was addressed in 7 of the 13 studies [19,21,22,32,33,35,37], while one article's reporting on intra-rater reliability was unclear [23]. Three articles reported inter-rater reliability [19,22,33]. Based on criteria on randomization and reliability testing, none of the studies scored low for risk of bias. Five studies were categorized as having a medium risk of bias [19,20,22,32,33], while the remaining eight were deemed to have a high overall risk of bias [21,23,34,35,36,37,38,39].

### 3.3. Demographic Data

The main characteristics of the included studies are summarized in Table 3. Five [19,21,22,32,38] of the studies were prospective, five [33,35,36,37,39] were retrospective, and three [20,23,34] had an unclear study type. A total of 11 studies evaluated maxillary molar distalization [19,20,21,22,23,32,33,34,35,36,37]. Wu et al. [38] and Rota et al. [39] were the only studies that evaluated mandibular molar distalization. Most of the studies utilized Invisalign as their choice of clear aligner, but an article by Zhang et al. [23] used Angel Aligner and an article by D’Anto et al. [19] used Ordoline aligners. Articles by Li et al. [34] and Cui et al. [36] did not identify what clear aligner brands were used.

In terms of the type and timing of treatment records, six studies (five for maxillary molar distalization [19,20,21,22,23] and one for mandibular molar distalization [38]) evaluated post-distalization records, and seven studies (six for maxillary molar distalization [32,33,34,35,36,37,68] and one for mandibular molar distalization [39]) evaluated post-treatment records. Within each time point, a mixture of dental model, lateral cephalometric analysis, and CBCT analysis were presented among the studies. Thus, the data collection and analysis were sub-grouped based on the arch in which distalization was performed as well as the timing and type of records provided in each included article.

### 3.4. Maxillary Molar Distalization

The amount of maxillary molar distalization was evaluated on post-treatment and post-distalization dental models (Table 4) and post-treatment radiographs (Table 5). Distalization was measured based on varying parameters.

**Figure 2:**
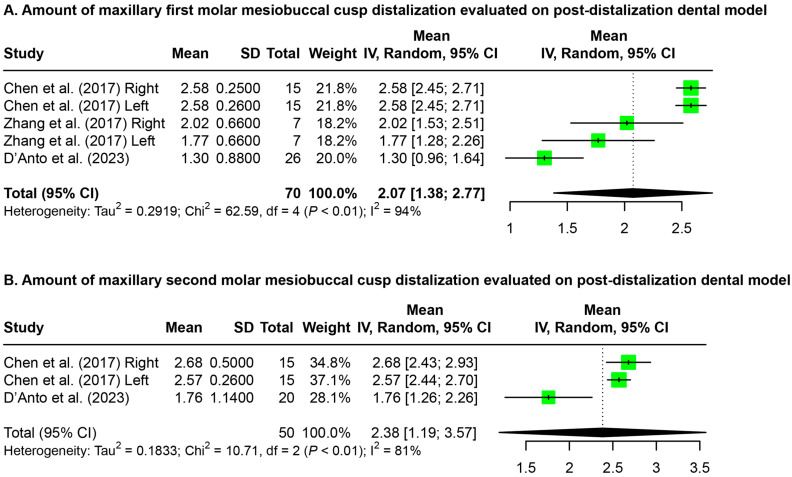
Forest plots for the amount of maxillary molar distalization evaluated on the dental model. SD: standard deviation; CI: confidence interval [19,20,23].

Meta-analyses (Figure 2) were performed on the amount of maxillary first and second molar mesiobuccal cusp distalization evaluated on post-distalization dental models. The maxillary first and second molars showed significant distalization of 2.07 mm [1.38 mm, 2.77 mm] and 2.38 mm [1.19 mm, 3.57 mm], respectively.

**Figure 3:**
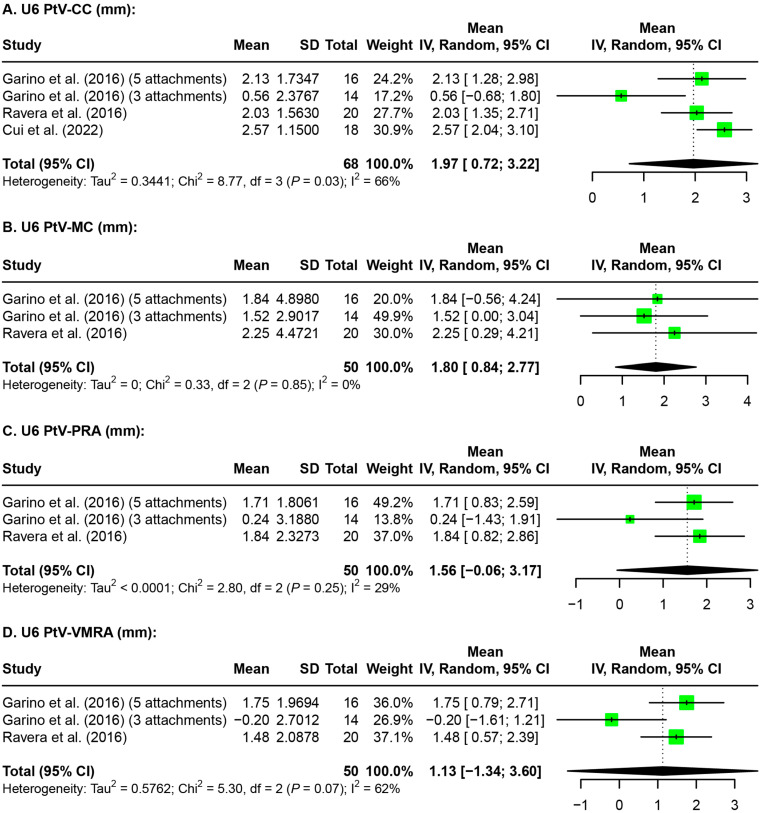
Forest plots for the amount of maxillary first molar distalization evaluated on the radiographic images. U6: maxillary first molar; PtV-CC: distance between the pterygoid vertical plane and the center of the crown of the maxillary molar; PtV-MC: distance between the pterygoid vertical plane and the mesial cusp of the maxillary molar; PtV-PRA: distance between the pterygoid vertical plane and the palatal root apex of the maxillary molar; PtV-VMRA: distance between the pterygoid vertical plane and the vestibulomesial root apex of the maxillary molar; SD: standard deviation; CI: confidence interval [32,33,36].

**Figure 4:**
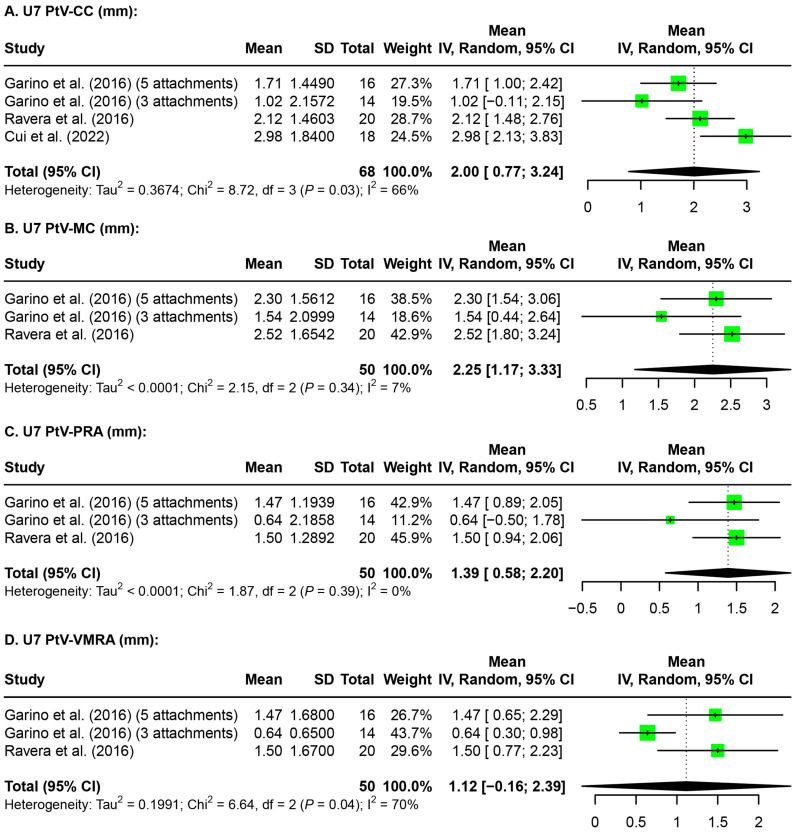
Forest plots for the amount of maxillary second molar distalization evaluated on the radiographic images. U7: maxillary second molar; PtV-CC: distance between the pterygoid vertical plane and the center of the crown of the maxillary molar; PtV-MC: distance between the pterygoid vertical plane and the mesial cusp of the maxillary molar; PtV-PRA: distance between the pterygoid vertical plane and the palatal root apex of the maxillary molar; PtV-VMRA: distance between the pterygoid vertical plane and the vestibulomesial root apex of the maxillary molar; SD: standard deviation; CI: confidence interval [32,33,36].

Meta-analyses were also performed on the amount of maxillary first (U6, Figure 3) and second molar (U7, Figure 4) distalization evaluated on radiographic images. A random effects model was used for meta-analysis for the parameters U6 and U7 PtV-CC (distance between the pterygoid vertical plane and the center of the crown of the maxillary molar), PtV-MC (distance between the pterygoid vertical plane and the mesial cusp of maxillary molar), PtV-PRA (distance between the pterygoid vertical plane and the palatal root apex of the maxillary molar), and PtV-VMRA (distance between the pterygoid vertical plane and the vestibulomesial root apex of maxillary molar). Of the four parameters analyzed for the U6 distalization evaluated on radiographic images, both parameters at the crown level showed a significant amount of distalization, while both parameters at the root level showed non-significant distalization (Figure 3). Of the four parameters analyzed for U7 distalization evaluated on radiographic images, parameters at the crown level as well as at the palatal root apex showed a significant amount of distalization, while the mesial buccal root apex did not (Figure 4).

### 3.5. Maxillary Molar Rotation and Tipping

Maxillary molar rotation accompanying maxillary molar distalization was only evaluated on post-distalization and post-treatment dental models (Table 6). Maxillary molar tipping accompanying maxillary molar distalization was evaluated on post-treatment radiograph images and dental models (Table 7).

**Figure 5:**
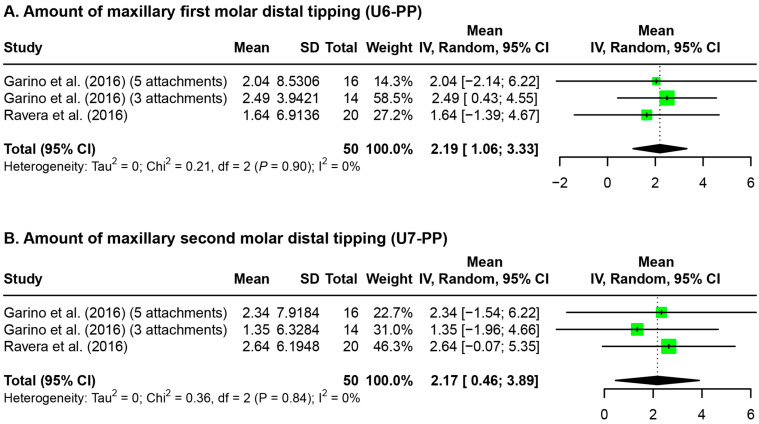
Forest plot for the amount of maxillary molar distal tipping after maxillary molar distalization. U6-PP: maxillary first molar–palatal plane angle; U7-PP: maxillary second molar–palatal plane angle; SD: standard deviation; CI: confidence interval [32,33].

Due to the high heterogeneity of the parameters reported in the included studies, a meta-analysis could only be performed on the amount of maxillary first and second molar distal tipping in reference to the palatal plane on post-treatment radiographic images (Figure 5). The analysis revealed 2.19° [1.06°, 3.33°] of crown distal tipping of U6s and 2.17° [0.46°, 3.89°] of crown distal tipping of U7s.

### 3.6. Anterior Anchorage Loss after Maxillary Molar Distalization

The amount of anchorage loss on both upper and lower arches during maxillary molar distalization was evaluated on both dental models (Table 8) and radiographic images (Table 9).

**Figure 6:**
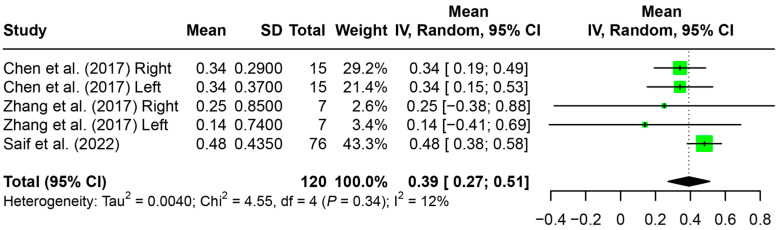
Forest plot for the amount of maxillary central incisor protrusion after maxillary molar distalization based on the evaluation of pre-treatment and post-distalization dental models. SD: standard deviation; CI: confidence interval [20,22,23].

**Figure 7:**
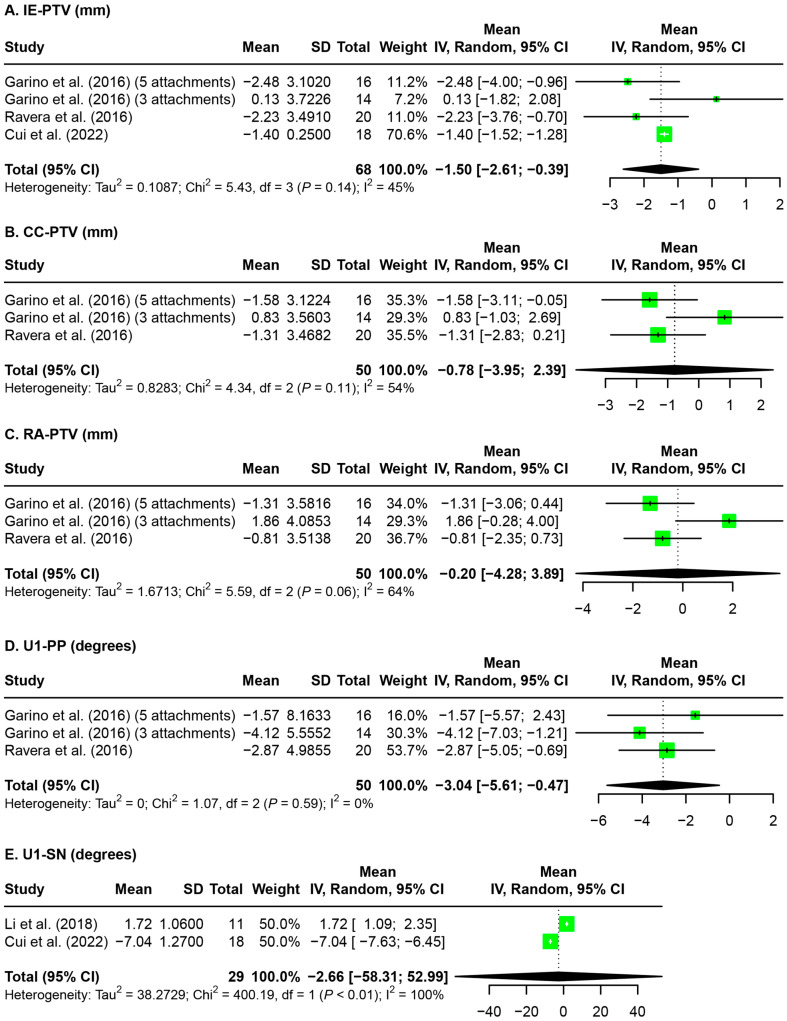
Forest plot for the amount of maxillary central incisor protrusion and proclination after maxillary molar distalization based on the evaluation of pre- and post-treatment radiographic analysis. IE-PTV: incisal edge–pterygoid vertical; CC-PTV: center of crown–pterygoid vertical; RA-PTV: root apex–pterygoid vertical; U1-PP: upper incisor–palatal plane angle; U1-SN: upper incisor–sella-nasion angle; SD: standard deviation; CI: confidence interval [32,33,34,36].

Meta-analyses were conducted on the amount of maxillary central incisor protrusion based on the evaluation of pre-treatment and post-distalization dental models (Figure 6) as well as on the amount of maxillary central incisor protrusion and proclination based on the evaluation of pre- and post-treatment radiographic analysis (Figure 7). At the post-distalization time point, 0.39 mm [0.27 mm, 0.51 mm] of maxillary central incisor protrusion was observed (Figure 6). At the post-treatment time point, 1.50 mm [0.39 mm, 2.61 mm] of central incisor retraction at the incisal edge level (IE-PTV) was observed, while no significant amount of retraction was observed of the central incisor at the crown level (CC-PTV, −0.78 mm [−3.95 mm, 2.39 mm]) or at the root apex level (RA-PTV, −0.20 mm [−4.28 mm, 3.89 mm]). Slight incisal retroclination was observed based on the U1-PP angle (−3.40° [−5.61°, −0.47°]), but not based on the U1-SN angle (−2.66° [−58.31°, 52.99°]) (Figure 7). Meta-analyses could not be performed on the opposing arch evaluations due to insufficient data.

### 3.7. Mandibular Molar Distalization

The amount of mandibular molar distalization (Table 10), mandibular molar tipping (Table 11), and anterior anchorage loss after mandibular molar distalization (Table 12) was evaluated on post-distalization and post-treatment radiographic images with one study available from each time point. Further analysis of mandibular molar distalization with a clear aligner sequential distalization protocol was not possible due to insufficient data.

## 4. Discussion

### 4.1. Summary of Evidence

Molar distalization has been a topic of ongoing debate in orthodontics. With the increase in popularity of clear aligners in the past two decades, whether clear aligners can effectively achieve a significant amount of molar distalization with the sequential distalization protocol has become a hot topic of discussion. Due to the varying evidence supporting the efficiency of molar distalization with clear aligners, this study further elucidates currently available data on clear aligner molar distalization and the accompanying side effects.

Our literature search showed high variability in study characteristics, limiting the data that could be utilized for each meta-analysis. Overall, about 2 mm of maxillary molar crown distalization was observed, accompanied by molar crown distal tipping and intra-arch anterior anchorage loss. There was not enough evidence on inter-arch anchorage loss, as well as on mandibular molar distalization with clear aligners. In addition, large variations in the amount of achieved molar distalization and anterior teeth movement were noticed among the reported studies.

Discrepancies between the distalization of the maxillary molars achieved by each study can be attributed to several factors. Primarily, differences in the time points may not be comparable due to changes in molar position during orthodontic treatment between the post-distalization and post-treatment stages. Additionally, studies contained data that varied greatly in the types of records, parameters, and time points, making the available data difficult to compare. Even within the same studies, variations were found in the amount of distalization achieved when different structures and measurement parameters were used (Figure 2, Figure 3 and Figure 4). Tracing errors due to distortion, differences in magnification, and overlapping structures on radiographic image superimpositions were also important contributing factors in the variations in molar distalization determined in this study.

The varying attachment designs of each study may also have affected the amount of distalization achieved. Garino et al. [32] noted a significant difference in the distalization achieved with the three-attachment protocol compared to the five-attachment protocol, with approximately 1.54 mm and 2.3 mm of maxillary first molar distalization achieved, respectively. Though the attachment protocol could potentially influence the efficiency of molar distalization, most studies did not include specific information regarding the attachment design. This lack of crucial information may have contributed to the variance in reported molar distalization from the included studies, further challenging the analysis of the available literature.

It is worth noting that none of the included studies compared clear aligner therapy to other well-studied molar distalization strategies. Thus, to obtain a better sense of the efficiency of molar distalization with clear aligners in comparison with fixed appliances, we can only compare the meta-analysis results with articles that report molar distalization with fixed appliances in adult patients. With a sample population of 33 adult patients that underwent molar distalization therapy using different types of intraoral distalizing appliances, including Pendulum, Distal Jet, and Fast Back appliances, 2.9 ± 0.6 mm maxillary first molar distalization was observed on post-treatment lateral cephalometric radiographs without significant molar crown distal tipping (U6-SN angle: −0.2° ± 1.8°) [69]. In another study evaluating 46 non-growing patients treated with different types of distalizing appliances (Cetlin distalizing appliance, compressed Niti coil springs, Loca system wire, intraoral palatal distalizing appliances, and “Zig-Zag loops” in conjunction with intermaxillary elastics), 2.16 ± 0.84 mm maxillary first molar distalization was observed on post-treatment lateral cephalometric radiographs accompanied with 1.45° (range 2.22° to −6.45°) of molar crown distal tipping [70]. Thus, the sequential distalization protocol of clear aligners appears to provide a slightly reduced amount of maxillary molar distalization (2.07 mm [1.38 mm, 2.77 mm]) with more prominent molar crown distal tipping (2.19° [1.06°, 3.33°]) in adult patients when compared to non-TAD-supported fixed appliances. However, additional studies directly comparing different treatment strategies are needed to provide clear evidence on this aspect.

Regarding the anterior anchorage loss during molar distalization, our study found significant maxillary incisor protrusion at the post-distalization time point, but incisor retraction and retroclination at the end of treatment. However, it is unclear if the space achieved for retraction was due purely to whole arch distalization or if the retraction space was from interproximal reduction or arch expansion. Future studies should provide more details on the specific protocols for a more accurate comparison of retraction achieved following molar distalization. In addition, although there was insufficient data to run a meta-analysis on the mandibular incisor sagittal position changes, the available data consistently show proclination and protrusion of lower incisors at post-distalization and post-treatment time points (Table 8 and Table 9), indicating significant mandibular anchorage loss during maxillary molar distalization with clear aligners.

### 4.2. Limitations

A major limitation of this study was the variation in the evaluation time points, the type of data collected, and the measurement parameters, which significantly affected the number of studies that could be incorporated into the meta-analysis. While both maxillary and mandibular molar distalization are pertinent in orthodontic treatment, only two of the 13 included studies included information regarding mandibular molar distalization [38,39]. Despite the inclusion of these two studies, mandibular molar measurements could not be analyzed via meta-analysis.

Secondly, none of the included studies provided details regarding the ClinCheck^®^ set-ups. Studies may have had differing amounts of molar distalization programmed into the digital set-up, which could affect the amount of molar distalization achieved clinically.

Thirdly, this study did not explore the vertical control and transverse expansion aspects of molar distalization. Further studies exploring all aspects of molar distalization with clear aligners are necessary to better support the clinical use of clear aligners in molar distalization.

Nevertheless, the current study points to the glaring gaps in the available data on molar distalization with clear aligners. More comprehensive studies in molar distalization by clear aligners and the accompanying effects are recommended.

## 5. Conclusions

This study demonstrates that approximately 2 mm maxillary molar distalization is achievable with the sequential distalization protocol of clear aligners with a certain amount of crown distal tipping. However, the high risk of bias among current available studies and the high variations in the time points assessed, type of data collected, and parameters measured among the available studies point to the insufficient data currently available on molar distalization with clear aligners. Additional studies are needed to determine if a sequential distalization protocol with clear aligners alone is a viable option for molar distalization.

## Figures and Tables

**Table 1:** The PICO questions of this study.

Criteria	Description
Population	Patients undergoing orthodontic treatment with clear aligners requiring molar distalization
Intervention	Molar distalization with sequential distalization protocol of clear aligner therapy
Comparisons	The control is pre-treatment models and radiographs
Outcome	The amount of molar distalization, molar rotation, molar tipping, and anterior anchorage loss achieved during molar distalization with clear aligners

**Table 2:** Risk of bias assessment of the thirteen included studies. ⊕: Low risk of bias; ?: medium risk of bias; ⊖: high risk of bias.

		Maxillary											Mandibular	
		Garino et al. (2016) [32]	Ravera et al. (2016) [33]	Chen et al. (2017) [20]	Zhang et al. (2017) [23]	Li et al. (2018) [34]	Caruso et al. (2019) [35]	Cui et al. (2022) [36]	Saif et al. (2022) [22]	D’Anto et al. (2023) [19]	Lin et al. (2023) [37]	Loberto et al. (2023) [21]	Wu et al. (2021) [38]	Rota et al. (2022) [39]
1. Study Design (6)	A. Objective: objective clearly formulated	⊕	⊕	⊕	⊕	⊕	⊕	⊕	⊕	⊕	⊕	⊕	⊕	⊕
	B. Sample size: considered adequate and estimated before collection of data	⊕	⊕	?	⊖	?	?	?	⊕	?	⊖	⊕	?	⊕
	C. Baseline characteristics: similar baseline characteristics	⊕	⊕	?	?	⊕	?	⊕	⊕	⊕	⊕	⊕	⊕	⊕
	D. Co-interventions	⊕	⊕	⊕	⊕	⊕	⊕	⊕	?	⊕	⊕	⊕	⊕	⊕
	E. Randomization													
	Random sampling	⊕	⊖	⊖	⊖	⊖	⊖	⊖	⊖	⊖	⊖	⊖	⊖	⊖
	Random allocation of treatment	⊕	⊖	⊖	⊖	⊖	⊖	⊖	⊖	⊖	⊖	⊖	⊖	⊖
2. Study Measurements (5)	F. Measurement method: appropriate to the objective	⊕	⊕	⊕	⊕	?	⊕	⊕	⊕	⊕	⊕	⊕	⊕	⊕
	G. Blind measurement: blinding													
	Blinding (examiner)	⊕	⊕	⊖	⊖	⊖	⊕	⊖	⊖	⊖	⊖	⊖	⊖	⊕
	Blinding (statistician)	⊕	⊖	⊖	⊖	⊖	⊖	⊖	⊖	⊖	⊖	⊖	⊖	⊖
	H. Reliability													
	Reliability described? (intra-rater reliability)	⊕	⊕	⊖	?	⊖	⊕	⊖	⊕	⊕	⊕	⊕	⊖	⊖
	Adequate level of agreement? (inter-rater reliability)	⊖	⊕	⊖	⊖	⊖	⊖	⊖	⊕	⊕	⊖	⊖	⊖	⊖
3. Statistical Analysis (5)	I. Statistical analysis													
	Appropriate for data	⊕	⊕	⊕	⊖	⊖	⊕	⊕	⊕	⊕	⊕	⊕	⊖	⊕
	Combined subgroup analysis	⊕	⊕	⊕	⊖	⊖	⊕	⊕	⊕	⊕	⊕	⊕	⊖	⊕
	J. Cofounders (co-interventions): confounders included in the analysis	⊕	⊕	⊖	⊖	⊖	⊕	⊕	?	⊕	⊕	⊕	⊖	⊕
	K. Statistical significance level													
	*p*-value stated?	⊕	⊕	⊕	⊖	⊕	⊕	⊕	⊕	⊕	⊕	⊕	⊕	⊕
	Confidence intervals stated?	⊖	⊕	⊖	⊖	⊖	⊖	⊖	⊖	⊕	⊖	⊕	⊖	⊕
4. Other	L. Clinical significance	⊕	⊕	⊕	?	⊕	⊕	⊕	⊕	⊕	⊕	⊕	⊕	⊕
	Total score	15	14	7	3	5	10	9	10	12	10	12	6	12
	Percentage of the total	88.24	82.35	41.18	17.65	29.41	58.82	52.94	58.82	70.59	58.82	70.59	35.29	70.59
Risk of bias		MED	MED	MED	HIGH	HIGH	HIGH	HIGH	MED	MED	HIGH	HIGH	HIGH	HIGH

**Table 3:** Characteristics of the thirteen included studies. F: female, M: male, CBCT: cone–beam computed tomography, Y: yes, N: no.

Study	Study Type	Maxillary or Mandibular	Patient Age (Years)	Sample Size (F/M)	Clear Aligner Brand	Post-Distalization Records			Post-Treatment Records		
						Digital Model	Lateral Ceph	CBCT	Digital Model	Lateral Ceph	CBCT
Garino et al. (2016) [32]	Prospective	Maxillary	30.5	30 (18F/12M)	Invisalign	-	-	-	N	Y	N
Ravera et al. (2016) [33]	Retrospective	Maxillary	29.73 ± 6.89	20 (11F/8M)	Invisalign	-	-	-	N	Y	N
Chen et al. (2017) [20]	Unclear	Maxillary	25.3 (14–43)	15	Invisalign	Y	N	N	-	-	-
Zhang et al. (2017) [23]	Unclear	Maxillary	14.0 ± 3.1	7 (5F/2M)	Angel Aligner	Y	N	N	-	-	-
Li et al. (2018) [34]	Unclear	Maxillary	25.3 (21–34)	11 (7F/4M)	Unclear	-	-	-	Y	Y	N
Caruso et al. (2019) [35]	Retrospective	Maxillary	22.7 ± 5.3	10 (8F/2M)	Invisalign	-	-	-	N	Y	N
Cui et al. (2022) [36]	Retrospective	Maxillary	27.8 ± 5.38 (18–38)	18	Unclear	-	-	-	N	N	Y
Saif et al. (2022) [22]	Prospective	Maxillary	25.4 (17–39)	38 (34F/4M)	Invisalign	Y	N	N	-	-	-
D’Anto et al. (2023) [19]	Prospective	Maxillary	25.7 ± 8.8 (18–45.5)	16 (12F/4M)	Ordoline aligner	Y	N	N	-	-	-
Lin et al. (2023) [37]	Retrospective	Maxillary	26.64 ± 3.02 (23.1–31.5)	7	Invisalign	-	-	-	Y	N	Y
Loberto et al. (2023) [21]	Prospective	Maxillary	14.9 ± 6	49 (27F/22M)	Invisalign	Y	N	N	-	-	-
Wu et al. (2021) [38]	Prospective	Mandibular	>18	20 (12F/8M)	Invisalign	N	N	Y	-	-	-
Rota et al. (2022) [39]	Retrospective	Mandibular	25.6 ± 4.5	16 (8F/8M)	Invisalign	-	-	-	N	Y	N

**Table 4:** The amount of maxillary molar distalization in millimeters (mm) evaluated on the dental model. The data are reported as mean ± standard deviation.

Time Points	Tooth	Parameters	References	Amount of Distalization
Post-distalization	U6 ^1^	MB ^3^ cusp (mm)	Chen et al. (2017) Right [20]	2.58 ± 0.25
			Chen et al. (2017) Left [20]	2.58 ± 0.26
			Zhang et al. (2017) Right [23]	2.02 ± 0.66 *
			Zhang et al. (2017) Left [23]	1.77 ± 0.66 *
			D’Anto et al. (2023) [19]	1.30 ± 0.88
			Loberto et al. (2023) Right [21]	2.4
			Loberto et al. (2023) Left [21]	2.4
		DB ^4^ cusp (mm)	D’Anto et al. (2023) [19]	1.42 ± 0.94
			Loberto et al. (2023) Right [21]	3.0
			Loberto et al. (2023) Left [21]	2.2
		CC ^5^ (mm)	Saif et al. (2022) [22]	1.81 ± 0.84
	U7 ^2^	MB cusp (mm)	Chen et al. (2017) Right [20]	2.68 ± 0.50
			Chen et al. (2017) Left [20]	2.57 ± 0.26
			D’Anto et al. (2023) [19]	1.76 ± 1.14
		DB cusp (mm)	D’Anto et al. (2023) [19]	1.54 ± 1.13
		CC (mm)	Saif et al. (2022) [22]	1.85 ± 0.88
Post-treatment	U6	Molar sagittal distance (mm)	Li et al. (2018) [34]	2.12 ± 1.09
		MB cusp (mm)	Lin et al. (2023) [37]	0.67 ± 0.50 ^$^
		DB cusp (mm)	Lin et al. (2023) [37]	0.84 ± 0.60 ^$^
		MP cusp ^6^ (mm)	Lin et al. (2023) [37]	0.36 ± 0.40 ^$^

^1^ U6: maxillary first molar; ^2^ U7: maxillary second molar; ^3^ MB: mesiobuccal; ^4^ DB: distobuccal; ^5^ CC: center of crown; ^6^ MP: mesiopalatal; *: average and standard deviation calculated based on data provided on the distalization of maxillary molars obtained from seven patients; ^$^: data from a dental model incorporated into CBCT.

**Table 5:** The amount of maxillary molar distalization in millimeters (mm) was evaluated on the radiographic images. The data are reported as either mean ± standard deviation or mean [95% confidence interval].

Time Points	Tooth	Parameters	References	Amount of Distalization
Post-treatment	U6 ^1^	PtV-CC ^3^ (mm)	Garino et al. (2016) (5 attachments) [32]	2.13 [1.28, 2.98]
			Garino et al. (2016) (3 attachments) [32]	0.56 [−0.68, 1.81]
			Ravera et al. (2016) [33]	2.03 [1.35, 2.72]
			Cui et al. (2022) [36]	2.57 ± 1.15 *
		PtV-MC ^4^ (mm)	Garino et al. (2016) (5 attachments) [32]	1.84 [−0.56, 4.24]
			Garino et al. (2016) (3 attachments) [32]	1.52 [−0.07, 2.97]
			Ravera et al. (2016) [33]	2.25 [0.29, 4.21]
		PtV-PRA ^5^ (mm)	Garino et al. (2016) (5 attachments) [32]	1.71 [0.83, 2.60]
			Garino et al. (2016) (3 attachments) [32]	0.24 [−1.43, 1.91]
			Ravera et al. (2016) [33]	1.84 [0.82, 2.86]
		PtV-VMRA ^6^ (mm)	Garino et al. (2016) (5 attachments) [32]	1.75 [0.78, 2.71]
			Garino et al. (2016) (3 attachments) [32]	−0.20 [−1.62, 1.21]
			Ravera et al. (2016) [33]	1.48 [0.57, 2.40]
		UMD ^7^ (mm)	Li et al. (2018) [34]	2.32 ± 0.84
	U7 ^2^	PtV-CC (mm)	Garino et al. (2016) (5 attachments) [32]	1.71 [1.00, 2.42]
			Garino et al. (2016) (3 attachments) [32]	1.02 [−0.11, 2.15]
			Ravera et al. (2016) [33]	2.12 [1.48, 2.76]
			Cui et al. (2022) [36]	2.98 ± 1.84 *
		PtV-MC (mm)	Garino et al. (2016) (5 attachments) [32]	2.30 [1.53, 3.06]
			Garino et al. (2016) (3 attachments) [32]	1.54 [0.44, 2.64]
			Ravera et al. (2016) [33]	2.52 [1.79, 3.24]
		PtV-PRA (mm)	Garino et al. (2016) (5 attachments) [32]	1.47 [0.89, 2.06]
			Garino et al. (2016) (3 attachments) [32]	0.64 [−0.51, 1.78]
			Ravera et al. (2016) [33]	1.50 [0.94, 2.07]
		PtV-VMRA (mm)	Garino et al. (2016) (5 attachments) [32]	1.68 [1.10, 2.25]
			Garino et al. (2016) (3 attachments) [32]	0.65 [−0.55, 1.86]
			Ravera et al. (2016) [33]	1.67 [1.03, 2.31]

^1^ U6: maxillary first molar; ^2^ U7: maxillary second molar; ^3^ PtV-CC: distance between the pterygoid vertical plane and the center of the crown of the maxillary molar; ^4^ PtV-MC: distance between the pterygoid vertical plane and the mesial cusp of the maxillary molar; ^5^ PtV-PRA: distance between the pterygoid vertical plane and the palatal root apex of the maxillary molar; ^6^ PtV-VMRA: distance between the pterygoid vertical plane and the vestibulomesial root apex of the maxillary molar; ^7^ UMD: upper molar distalization; *: data from lateral cephalometric radiograph extracted from CBCT.

**Table 6:** The amount of maxillary molar rotation in degrees (°) during maxillary molar distalization was evaluated on the dental model. The data are reported as mean ± standard deviation.

Time Points	Tooth	Parameters	References	Distal In–Mesial out Rotation
Post-distalization	U6 ^1^	MB-DP ^3^ (°)	Chen et al. (2017) Right [20]	0.93 ± 3.51
			Chen et al. (2017) Left [20]	0.75 ± 1.74
		DB-MP ^4^ (°)	D’Anto et al. (2023) [19]	8.09 ± 4.80
	U7 ^2^	MB-DP (°)	Chen et al. (2017) Right [20]	0.85 ± 2.20
			Chen et al. (2017) Left [20]	0.86 ± 2.16
		DB-MP (°)	D’Anto et al. (2023) [19]	6.40 ± 4.14
Post-treatment	U6	Molar rotation (°)	Li et al. (2018) [34]	3.77 ± 4.88

^1^ U6: maxillary first molar; ^2^ U7: maxillary second molar; ^3^ MB-DP: mesiobuccal cusp–distopalatal cusp displacement angulation; ^4^ DB-MP: distobuccal cusp–mesiopalatal cusp displacement angulation.

**Table 7:** The amount of maxillary molar tipping in degrees (°) during maxillary molar distalization. The data are reported as either mean ± standard deviation or mean [95% confidence interval].

Time Points	Tooth	Parameters	References	Distal Tipping
Post-treatment	U6 ^1^	UM-SN ^3^ (°)	Li et al. (2018) [34]	3.83 ± 1.37
		U6-SN ^4^ (°)	Cui et al. (2022) [36]	3.43 ± 2.71 *
		U6-PP ^5^ (°)	Garino et al. (2016) (5 attachments) [32]	2.04 [−2.14, 6.22]
			Garino et al. (2016) (3 attachments) [32]	2.49 [0.42, 4.55]
			Ravera et al. (2016) [33]	1.64 [−1.39, 4.67]
			Caruso et al. (2019) [35]	1.3 ^&^
		Distal tipping (evaluated on the dental model, °)	Zhang et al. (2017) Right [23]	5.70 ± 3.03 ^$^
			Zhang et al. (2017) Left [23]	4.09 ± 3.30 ^$^
	U7 ^2^	U7-SN ^6^ (°)	Cui et al. (2022) [36]	4.34 ± 3.28 *
		U7-PP ^7^ (°)	Garino et al. (2016) (5 attachments) [32]	2.34 [−1.54, 6.22]
			Garino et al. (2016) (3 attachments) [32]	1.35 [−1.97, 4.66]
			Ravera et al. (2016) [33]	2.64 [−0.06, 5.37]
			Caruso et al. (2019) [35]	−0.6 ^&^

^1^ U6: maxillary first molar; ^2^ U7: maxillary second molar; ^3^ UM-SN: maxillary molar sella-nasion angle; ^4^ U6-SN: maxillary first molar sella-nasion angle; ^5^ U6-PP: maxillary first molar palatal plane angle; ^6^ U7-SN: maxillary second molar sella-nasion angle; ^7^ U7-PP: maxillary second molar palatal plane angle; *: data from lateral cephalometric radiograph extracted from CBCT; ^&^: data calculated based on post-treatment mean value—pre-treatment mean value provided in the article; ^$^: average and standard deviation calculated based on the individual data of seven patients.

**Table 8:** The amount of anchorage loss during maxillary molar distalization was evaluated on the dental model. A positive value indicates the protrusion and proclination of the anterior teeth. The data are reported as mean ± standard deviation.

Time Points	Arch	Parameters	References	Amount of Change
Post-distalization	Same arch	U1 ^1^ protrusion (mm)	Chen et al. (2017) Right [20]	0.34 ± 0.29
			Chen et al. (2017) Left [20]	0.34 ± 0.37
			Zhang et al. (2017) Right [23]	0.25 ± 0.85 *
			Zhang et al. (2017) Left [23]	0.14 ± 0.74 *
			Saif et al. (2022) [22]	0.48 ± 0.435
		U2 ^2^ protrusion (mm)	Saif et al. (2022) [22]	0.45 ± 0.384
		U3 ^3^ mesialization (mm)	Loberto et al. (2023) Right [21]	1.5
			Loberto et al. (2023) Left [21]	1.15
			Saif et al. (2022) [22]	0.27 ± 0.250
		U1 proclination (°)	Zhang et al. (2017) Right [23]	−1.94 ± 2.61 *
			Zhang et al. (2017) Left [23]	−1.02 ± 2.55 *
Post-treatment	Opposing arch	L1s ^4^ incisal edge (mm)	Lin et al. (2023) [37]	1.21 ± 0.86 ^&^
	Same arch	U1 incisal edge (mm)	Lin et al. (2023) [37]	−0.81 ± 0.89 ^&^

^1^ U1: maxillary central incisor; ^2^ U2: maxillary lateral incisor; ^3^ U3: maxillary canine; ^4^ L1s: mandibular central incisors; *: average and standard deviation calculated based on the individual data of seven patients; ^&^: data from the model integrated into CBCT.

**Table 9:** The amount of anterior anchorage loss during maxillary molar distalization was evaluated on the radiographic images. A positive value indicates the protrusion and proclination of the anterior teeth.

Time Points	Arch	Parameters	References	Amount of Change
Post-treatment	Same arch	IE-PTV ^1^ (mm)	Garino et al. (2016) (5 attachments) [32]	−2.48 [−4.00, −0.96]
			Garino et al. (2016) (3 attachments) [32]	0.13 [−1.82, 2.08]
			Ravera et al. (2016) [33]	−2.23 [−3.76, −0.70]
			Cui et al. (2022) [36]	−1.40 ± 0.25 ^&^
		CC-PTV ^2^ (mm)	Garino et al. (2016) (5 attachments) [32]	−1.58 [−3.11, −0.05]
			Garino et al. (2016) (3 attachments) [32]	0.83 [−1.04, 2.69]
			Ravera et al. (2016) [33]	−1.31 [−2.83, 0.21]
		RA-PTV ^3^ (mm)	Garino et al. (2016) (5 attachments) [32]	−1.31 [−3.06, 0.45]
			Garino et al. (2016) (3 attachments) [32]	1.86 [−0.28, 4.00]
			Ravera et al. (2016) [33]	−0.81 [−2.35, 0.73]
		U1D ^4^ (mm)	Li et al. (2018) [34]	1.45 ± 0.94
		U1-PP ^5^ (°)	Garino et al. (2016) (5 attachments) [32]	−1.57 [−5.57, 2.43]
			Garino et al. (2016) (3 attachments) [32]	−4.12 [−7.03, −1.21]
			Ravera et al. (2016) [33]	−2.87 [−5.06, −0.69]
			Caruso et al. (2019) [35]	−13.5 *
		U1-SN ^6^ (°)	Li et al. (2018) [34]	1.72 ± 1.06
			Cui et al. (2022) [36]	−7.04 ± 1.27 ^&^
			Lin et al. (2023) [37]	−5.03 *
	Opposing arch	L1-MP ^7^ (°)	Li et al. (2018) [34]	3.82 ± 2.19
			Lin et al. (2023) [37]	6.57 *

^1^ IE-PTV: incisal edge–pterygoid vertical; ^2^ CC-PTV: center of crown–pterygoid vertical; ^3^ RA-PTV: root apex–pterygoid vertical; ^4^ U1D: upper incisor distalization; ^5^ U1-PP: upper incisor–palatal plane angle; ^6^ U1-SN: upper incisor–sella-nasion angle; ^7^ L1-MP: lower incisor–mandibular plane angle; *: data calculated based on the difference between the mean value of post-treatment incisor angulation and the mean value of pre-treatment incisor angulation; ^&^: data from CBCT.

**Table 10:** The amount of mandibular molar distalization in millimeters (mm) was evaluated on the radiographic images. The data are reported as either mean ± standard deviation or mean ± standard error (SE).

Time Points	Tooth	Parameters	References	Amount of Distalization
Post-distalization	L6 ^1^	MBC ^3^ (mm)	Wu et al. (2021) [38]	0.78 ± 0.33
		DBC ^4^ (mm)	Wu et al. (2021) [38]	0.91 ± 0.31
		MLC ^5^ (mm)	Wu et al. (2021) [38]	0.56 ± 0.89
		DLC ^6^ (mm)	Wu et al. (2021) [38]	0.62 ± 0.84
		MRA ^7^ (mm)	Wu et al. (2021) [38]	0.29 ± 1.08
		DRA ^8^ (mm)	Wu et al. (2021) [38]	0.28 ± 0.66
		CC ^9^ (mm)	Wu et al. (2021) [38]	0.53 ± 1.37
		RC ^10^ (mm)	Wu et al. (2021) [38]	0.41 ± 0.96
	L7 ^2^	MBC (mm)	Wu et al. (2021) [38]	0.81 ± 1.46
		DBC (mm)	Wu et al. (2021) [38]	1.06 ± 0.65
		MLC (mm)	Wu et al. (2021) [38]	0.64 ± 1.19
		DLC (mm)	Wu et al. (2021) [38]	0.72 ± 1.07
		MRA (mm)	Wu et al. (2021) [38]	0.30 ± 1.01
		DRA (mm)	Wu et al. (2021) [38]	0.27 ± 0.82
		CC (mm)	Wu et al. (2021) [38]	0.59 ± 0.94
		RC (mm)	Wu et al. (2021) [38]	0.56 ± 0.91
Post-treatment	L6	mc-CoGo ^11^ (mm)	Rota et al. (2022) [39]	1.07 ± 1.61 (SE)
		cc-CoGo ^12^ (mm)	Rota et al. (2022) [39]	1.16 ± 1.49 (SE)
		mra-CoGo ^13^ (mm)	Rota et al. (2022) [39]	0.15 ± 1.71 (SE)
	L7	mc-CoGo (mm)	Rota et al. (2022) [39]	1.79 ± 1.57 (SE)
		cc-CoGo (mm)	Rota et al. (2022) [39]	2.47 ± 1.48 (SE)
		mra-CoGo (mm)	Rota et al. (2022) [39]	0.85 ± 1.63 (SE)

^1^ L6: mandibular first molar; ^2^ L7: mandibular second molar; ^3^ MBC: mesiobuccal cusp; ^4^ DBC: distobuccal cusp; ^5^ MLC: mesiolingual cusp; ^6^ DLC: distolingual cusp, ^7^ MRA: mesial root apex; ^8^ DRA: distal root apex; ^9^ CC: center of crown; ^10^ RC: center of root; ^11^ mc-CoGo: mandibular left first molar mesial crown point–Condylion–Gonion line; ^12^ cc-CoGo: mandibular left first molar central occlusal point–Condylion–Gonion line; ^13^ mra-CoGo: mandibular left first molar mesial root apex point–Condylion–Gonion line.

**Table 11:** The amount of mandibular molar tipping in degrees (°) during mandibular molar distalization. The data are reported as either mean ± standard deviation or mean ± standard error (SE).

Time Points	Tooth	Parameters	References	Distal Tipping
Post-distalization	L6 ^1^	L6 inclination (°)	Wu et al. (2021) [38]	1.62 ± 1.06
	L7 ^2^	L7 inclination (°)	Wu et al. (2021) [38]	2.10 ± 1.74
Post-treatment	L6	ax-CoGo ^3^ (°)	Rota et al. (2022) [39]	4.56 ± 2.03 (SE)
		ax-GoMe ^4^ (°)	Rota et al. (2022) [39]	5.03 ± 1.59 (SE)
	L7	ax-CoGo (°)	Rota et al. (2022) [39]	4.56 ± 4.15 (SE)
		ax-GoMe (°)	Rota et al. (2022) [39]	4.47 ± 1.92 (SE)

^1^ L6: mandibular first molar; ^2^ L7: mandibular second molar; ^3^ ax-CoGo: mandibular left molar long axis and Condylion–Gonion line; ^4^ ax-GoMe: mandibular left molar long axis and Gonion–Menton line.

**Table 12:** The amount of anterior anchorage loss during mandibular molar distalization. A positive value indicates the protrusion and proclination of the incisor; a negative value indicates the retrusion and retroclination of the incisor. The data are reported as either mean ± standard deviation or mean ± standard error (SE).

Time Points	Arch	Parameters	References	Amount of Change
Post-distalization	Same arch	L1ie ^1^ (mm)	Wu et al. (2021) [38]	1.02 ± 0.80 *
		L1ra ^2^ (mm)	Wu et al. (2021) [38]	0.33 ± 1.24 *
		L1cc ^3^ (mm)	Wu et al. (2021) [38]	0.56 ± 0.59 *
		L1 ^4^ inclination (°)	Wu et al. (2021) [38]	1.51 ± 1.51 *
Post-treatment	Same arch	31im-CoGo ^5^ (mm)	Rota et al. (2022) [39]	−1.13 ± 1.52 (SE)
		31ax-CoGo ^6^ (°)	Rota et al. (2022) [39]	−4.18 ± 2.44 (SE)
		31ax-GoMe ^7^ (°)	Rota et al. (2022) [39]	−4.78 ± 2.19 (SE)

^1^ L1ie: mandibular incisor incisal edge; ^2^ L1ra: mandibular incisor root apex; ^3^ L1cc: mandibular incisor center of crown; ^4^ L1: mandibular incisor; ^5^ 31im-GoGo: incisal margin of mandibular left central incisor and Condylion–Gonion line; ^6^ 31ax-CoGo: angulation between mandibular left first central incisor long axis and Condylion–Gonion line; ^7^ 31ax-GoMe: angulation between mandibular left first central incisor long axis and Gonion–Menton line; *: data from CBCT.

## Data Availability

The original contributions presented in the study are included in the article; further inquiries can be directed to the corresponding author.
